# Anesthesia for COVID-19 patients: protocol and precautions

**DOI:** 10.11604/pamj.supp.2020.35.2.23365

**Published:** 2020-05-18

**Authors:** Ouissal Aissaoui, Afak Nsiri, Yassine Hafiani, Rachid Alharrar

**Affiliations:** 1 Anesthesiology and ICU Department, University Hospital of Casablanca, Morocco

**Keywords:** COVID-19, anesthesia, emergency procedures

## To the editors of the Pan African Medical Journal

Covid-19 pandemic is rapidly evolving through Africa. In Morocco, we count more than 5000 cases and more than 150 deaths to date. Although mortality rates are low, this disease is highly contagious [1]. It’s transmitted through droplets and medical staff performing aerosol generating procedures is at high risk of contamination. Anesthesiologists are mostly exposed as they perform these procedures such a as intubation, extubation, tracheal and oropharyngeal succioning. Although elective surgery is canceled in our university hospital, we still deal with emergency interventions in suspected COVID-19 patients. In these cases, emergency procedures must be performed and waiting for rt PCR results is not always possible. Therefore, we established a fast track specifically for suspected cases. We organized the procedure into 5 steps:

**Step 1: admission to emergency department:** the patient is admitted to the emergency department. In case of fever or respiratory symptoms, Chest CT scan, Blood tests and rt PCR are performed. Patients with suspected CT images are transferred to the COVID-19 dedicated Operating room.

**Figure 1:**
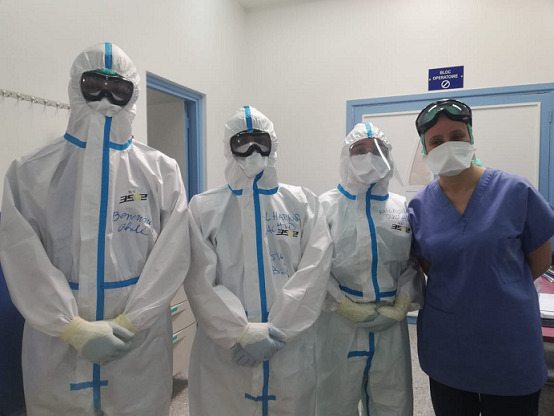
Aneshesiology team wearing PPE

**Step 2: transfert:** the patient’s transfer is coordinated by the emergency department’s team along with surgery and anesthesiology’s members. The patient wear a facial mask and is transferred in a COVID-19 dedicated ambulance. Staff accompanying the patient wear personal protective equipment (PPE) in accordance with the World health organization’s recommendations [2]. PPE comprised a FFP2 face mask, Protective cover all body suit or surgical hood, goggles or visor and double gloves. We have noticed that communication is made difficult because team members don’t recognize each other due to PPE. Therefore, we wrote staff names and roles on the body suits’ front and back (Figure 1).

**Step 3: anesthesia:** anesthesiology team is formed by a Senior Anesthesiology specialist managing the airway (preoxygenation, intubation, extubation), a Junior Anesthesiology specialist managing the ventilator’s settings during the procedure as well as an Anesthesiology Nurse, managing crush induction, antibioprophylaxy, and monitoring during general anesthesia. Prior to patient’s induction, we make sure that the high-efficiency hydrophobic filter is applied between the face mask and the breathing circuit and on the expiratory valve. We remove the patient’s facial mask and firmly apply preoxygenating mask. We start the ventilator and preoxygenation is applied during 5 minutes. Drugs are administrated according to crush induction protocol, in the following order: propofol 2 mg/Kg then Rocuronium 1mg/Kg. When the patient is apneic, the ventilator is stopped. Senior Anesthesiology specialist performs Intubation using a videolaryngoscope. Endotracheal tube (ET) is inserted and the cuff is inflated. The ventilator is connected and ventilation started. Protective ventilation is stared using the following parameters: Vt 6ml/Kg; FR 16 cycles per minute then adjusted to EtCO2; FiO2 necessary to obtain a pulse oxymetry > 92%; PEEP 6-8cm H2O; P plateau is maintained < 30cm H2O and driving pressure < 14.

**Step 4: surgery:** surgeons wear PPE and do not access the operating room until the patient is intubated and connected to the ventilator.

**Step 5: decision on COVID-19:** at the end of the intervention, rt-PCR results are recuperated and then transfer decision is made. COVID-19 patients are transferred to COVID-19 dedicated ICU and the operating room is sterilized. Negative patients are transferred to surgical ICU.

## Conclusion

We share our experience regarding suspected COVID-19 presenting with surgical emergencies. Adopting these procedures avoided delaying suspected or confirmed cases without exposing medical staff to contamination

## Competing interests

The author declares no competing interests.
